# Do Indian women know about and use the emergency contraceptive pill? An analysis of nationally representative data from 2005–06 and 2019–21

**DOI:** 10.1093/heapol/czad049

**Published:** 2023-11-16

**Authors:** Renu, Pooja Arora, Kerry Scott, Dina Balabanova

**Affiliations:** India Health Action Trust, Lucknow, Uttar Pradesh 226001, India; International Institute for Population Studies, Mumbai, Maharashtra 400088, India; Department of International Health, Johns Hopkins Bloomberg School of Public Health, 615 N. Wolfe Street, Baltimore MD 21218, United States; Independent Research Consultant, Toronto, Canada; Department of Global Health and Development, London School of Hygiene & Tropical Medicine, London, United Kingdom

**Keywords:** Pregnancy, knowledge, family planning, reproductive health

## Abstract

The emergency contraceptive pill (ECP) is a post-coital contraceptive method that prevents unintended pregnancy and is useful in specific circumstances. This study examined the awareness and use of the ECP in India, as there is scarce evidence in this area to guide policy development. This study used data from the 2005–06 (*n* = 124 385) and 2019–21 (*n* = 724 115) rounds of the National Family Health Survey of India. Bivariate analysis and multivariate logistic regression were applied to explore how demographic characteristics including age, education (none, primary, secondary, higher), wealth quintile, place of residence (urban, rural), marital status, parity, desire for children, whether current pregnancy was wanted, whether the women had had an abortion in the past 5 years, and whether current use of contraceptive methods affected the likelihood of knowledge and use of ECP. In the past 15 years, knowledge of ECP has increased by 37 percentage points but still remains relatively low at 48%. Less than 1% of the sample (0.55%) used ECP. Women aged 30–34 years, from wealthier and more educated backgrounds, living in urban areas, and currently using condoms had a higher likelihood of using ECP in comparison with women from age group 15–19 years, those from the poorest backgrounds, those with no education, those living in rural areas and those not using any contraceptive method, respectively. In comparison to married women, never-married women exhibited a higher awareness of ECP but lower use. More efforts must be made to improve awareness of the ECP, especially among adolescents, those with less education, poorer women and those in rural areas. The government is taking steps to improve access to ECP, and India’s female community health workers, the ASHAs, could be further supported to enhance awareness of ECP.

Key messages

- In 2019–21, more than half of Indian women were not aware of emergency contraceptive pills (ECP), and less than 1% had ever used an ECP, although this percentage has increased dramatically since 2005–06.
- ECP use is higher among younger and unmarried women, but awareness is lower compared to middle-aged and married women.
- Increasing awareness about the ECP, particularly among adolescents, those with less education, poorer women and those in rural areas, could reduce unintended pregnancy and expand women’s options for reproductive control.
- Measures to improve awareness and appropriate use of ECP could involve government frontline health workers, such as ASHA community health workers, but after providing them with more training and support to lead this effort.

## Introduction

Debates around contraception and population growth have had a long history. The World Population Conferences established in the 1920s involved discussions by policy makers and leaders on issues related to overpopulation in developing countries. The 1994 International Conference on Population and Development in Cairo, convened by the United Nations, went beyond giving recommendations for population control and addressed the intersection of reproductive rights and development and population policies needed across different sectors (UNFPA, 1994). The Cairo Declaration on Population & Development endorsed the concept of reproductive health, conceptualizing the role of family planning as part of a broader package of reproductive health care (UNFPA, 1994). Underlying this new paradigm was the proposition that enhancing individual health and reproductive control would ultimately lower fertility and slow population growth. The Cairo Conference also generated substantial discussion on abortion and the appropriate use of many well-known methods of contraception, including barrier methods, oral contraceptives and contraceptive implants (UNFPA, 1994).

However, emergency contraception has received less attention in international forums and agreements. Although emergency contraception is not designed as a primary form of contraception, widespread awareness of its existence and utility is vital to reduce the chance of pregnancy after unprotected sex or a failure of other birth control methods. There are two forms of emergency contraception: the emergency contraception pill (ECP) and the copper intrauterine device (Cu-IUD), both of which can be used to prevent pregnancy shortly after unprotected intercourse (WHO, 2021).

The ECP is often referred to as a morning-after or post-coital pill. The oldest form of post-coital ECP was developed in 1974 (Koyama *et al.*, 2013). ECPs are very effective at reducing the chances of pregnancy when taken within 72 hours of unprotected intercourse (WHO, 2021). Some studies have shown that taking an ECP minimizes the risk of pregnancy up to 120 hours after unprotected intercourse. Effectiveness is higher the sooner the ECP is used. Similarly, a Cu-IUD can be used within five days of unprotected intercourse (WHO, 2021).

Such post-coital hormonal treatments appear to prevent pregnancy by temporarily disrupting a woman’s hormonal patterns, thereby delaying ovulation, reducing the growth of the uterine lining, and preventing the fertilized ovum from travelling through the fallopian tubes (Conard and Gold, 2004; Clue, 2021). These disruptions are not permanent and last only a few days. Because the ECP does not interfere with the maintenance of a pregnancy after implantation, it is not considered an abortifacient. The ECP regimens recommended by WHO are ulipristal acetate, levonorgestrel, or combined oral contraceptives consisting of ethinyl oestradiol combined with levonorgestrel (WHO, 2021). Following the use of ECPs, women or girls may resume or initiate a regular method of contraception, but if a Cu-IUD is used for emergency contraception, no additional contraceptive protection is required subsequently (WHO, 2021).

The key benefit of using ECP is the prevention of unintended pregnancies after unprotected intercourse (including during sexual assault), incorrect use of contraceptives, and if there are concerns about possible contraceptive failure (NHM, 2008; WHO, 2021). Regular usage of ECP is not recommended because it is less effective than other forms of contraception, and its side effects (bleeding and nausea) can be unpleasant (Planned Parenthood, 2021). Yet widespread access to the ECP can prevent unintended pregnancy, thereby averting abortion (including unsafe abortion) or unwanted childbearing, which violates women’s reproductive freedoms and places her and the child’s physical and mental health in danger. Many unintended pregnancies result in unsafe abortions, which remain a significant contributor to maternal morbidity globally (Hajizadeh and Nghiem, 2020). In India, 15 · 6 million abortions occurred in 2015, resulting in an abortion rate of 47 per 1000 women aged 15–49 years (Singh *et al.*, 2018). Unintended pregnancies are also associated with delayed antenatal care initiation, higher maternal, neonatal and infant mortality rates, and poor child nutritional status (Hajizadeh and Nghiem, 2020). Unintended pregnancies in adolescents are especially detrimental, increasing vulnerability through limiting educational options, increasing welfare dependency and raising the likelihood of domestic violence (Malarcher *et al.*, 2010).

WHO recommends that national family planning programmes include the provision of emergency contraception, and complying with that, 59 countries include ECP in their essential medicine lists (ICEC, 2020). ECPs are available over the counter in over 60 countries, whereas the drug is available on prescription in 140 countries (ICEC, 2020). In some settings, ECP has faced challenges and resistance—the most common reason being the belief that it represents a form of abortion rather than a contraceptive (ICEC, 2020). A global review of knowledge and use of emergency contraception demonstrates wide variations across geographical regions. Awareness of emergency contraception among all women was found to be highest in Latin America (with a mean of 35%) and lowest in Asia (a mean of 11%) (Westley *et al.*, 2013). The same study also found that the prevalence of emergency contraception use among women who have ever had sex was highest in Latin America (3.5%), followed by Europe and West Asia (2.3%), Africa (1.8%) and East Asia (0.33%) (Westley *et al.*, 2013). More recently, a research study in Bangladesh established that among the 906 women in their nationally representative sample who had ever had an abortion or unwanted birth and had heard of the ECP, 99 women, which is 10.9% of the 906 ‘candidate’ women, had used ECP (Mahfuzur *et al.*, 2022). In a study of never-married adolescent girls aged 15–18 years in India using NFHS 2005–06 data, Narzary (2013) found that just 4.9% were aware of ECP. A small-scale study in an urban colony of Delhi found that 9% of 18–35-year-old married women had ever used the ECP (Singh *et al.*, 2015). A study among hospital staff in Chandigarh city in northern India found that 29 women (11.2%) were aware of ECP and only 3 women had ever used ECP out of the total sample of 258 women (Takkar *et al.*, 2005). A meta-analysis comprising 27 studies carried out over the past two decades in India, almost all of which were community-level and hospital-based studies and the majority of which were in northern states of India, found that the pooled proportion of women who ever used ECP was 6% (Mehta *et al.*, 2020).

The Drug Controller General of India permitted levonorgestrel as a dedicated product for emergency contraception in 2001 (NHM, 2008). Following this, ECP was introduced in 2003 by the Ministry of Health and Family Welfare (MoHFW) in India (NHM, 2008). In 2005, ECP was incorporated into the national family planning and rural health programmes, making it available at highly subsidized rates in rural areas (Shukla and Arends-Kuenning, 2017). Generally, the ECP is available through various outlets, including pharmacies, hospitals and private and public sector clinics—with wide variation across country contexts (ICEC, 2020). In India, ECP can be sold legally over the counter through a network of health-care facilities in both the public and private health sectors (NHM, 2008). In 2011, the MoHFW added ECP to the Accredited Social Health Activists’ (ASHA) drug kit. ASHAs are community outreach workers who focus on maternal, newborn and reproductive health (Dixit *et al.*, 2015).

Despite prioritizing sexual and reproductive health, unintended pregnancy continues to be a challenge in India. During 2015–19, 44% of all pregnancies in India were unintended, with 77% of those unintended pregnancies ending in abortion (Bearak *et al.*, 2022). Contraceptive prevalence is similarly low; according to the fifth National Family Health Survey (NFHS), the contraceptive prevalence rate (CPR) is 55% among women aged 15–49 years, and the unmet need for family planning is 9% (IIPS, 2021). Out of all unintended pregnancies occurring each year globally, more than one in seven are in India (State of the World Population Report, 2022). Despite institutional endorsement and relatively easy availability, awareness and use of ECP remain limited.

While recommending a move to modern contraceptive methods, international guidance suggests that there is a continued need for better information and provision of emergency contraception, particularly among low-income groups. This scenario is critical in countries characterized by large economic and social disparities, such as India. However, there is a shortage of analytical studies on the use and awareness of ECP and its socio-demographic and institutional determinants in India. The majority of the studies are small scale covering small geographical areas and we were only able to identify one national-level study within the past decade on ECP awareness and use in India (Narzary, 2013), and it was a study covering just adolescent girls. There is a need for research that covers all regions of India for the entire reproductive age population, so as to identify the target groups and regions on which the government could focus, when seeking to raise awareness of ECP. This study sought to fill these crucial gaps and explore the awareness of ECP in India and the levels and determinants of ECP use in order to arrive at policy recommendations that can facilitate the appropriate use of ECP.

## Methods

### Data source

We analysed data from two rounds of India’s NFHS (IIPS, 2021) to address the following research questions: (1) What is the level of awareness of ECP in India, and how does this vary across different groups/regions?; (2) How prevalent is the use of ECP, and how does this vary across different groups/regions?; (3) What is the association between repeated ECP use and the source of ECP?; and (4) How do these outcomes and associations vary over time?

The NFHS is a cross-sectional, nationally representative household survey that uses multi-stage stratified sampling conducted under theMoHFW, Government of India, by the nodal agency the International Institute for Population Sciences, Mumbai (IIPS, 2021). The NFHS has sought to strengthen India’s demographic and health data—providing vital data on health and family welfare and other related indicators at both the national and state levels, with five survey rounds completed until now. We have used data from NFHS-3, which was the first survey round since the implementation of ECP in India, and the latest round NFHS-5—undertaken in 2005–06 and 2019–21, respectively.

### Study participants

The study population comprises women aged 15–49 years from a representative sample of households across India (sample size: 124 385 in NFHS-3 and 724 115 in NFHS-5).

### Definition of variables

The NFHS defined emergency contraception as only pills rather than IUDs. This study analysed two outcomes of interest. The first outcome variable was awareness of ECP. Women were asked ‘Have you ever heard of emergency contraception? A woman can take pills up to three days after sexual intercourse to avoid becoming pregnant’. Women who replied ‘yes’ were considered aware and women who replied ‘no’ were considered unaware. The second outcome variable was whether the respondent had used ECP previously (‘ever ECP user’). The information on use of ECP was captured for ever-married women and sexually active unmarried women who ever had sexual intercourse. It was also analysed whether ECP was used twice or more in the past 12 months (‘frequent user’).

For factors affecting use and knowledge of ECP, necessary variables were identified according to their relevance to India and based on the global literature on ECP (Bayley *et al.*, 2009; Neustadt *et al.*, 2011; Alam *et al.*, 2020; Simmons *et al.*, 2022). For covariate selection in the multivariate analysis, chi-square test of association with *P* value < 0.05 and unadjusted odds ratio were employed amongst various socio-economic, demographic and background characteristics of the respondent in the bivariate analysis of the two outcome variables—awareness and use of ECP.

Socio-demographic variables included the woman’s age (categorized into 5-year age groups), place of residence, educational level, wealth index and religion, current marital status and region were used as independent variables. Regions comprised the different states/UTs of India. North region: Chandigarh, Delhi, Haryana, Himachal Pradesh, Jammu and Kashmir, Ladakh, Punjab, Rajasthan, Uttarakhand; central region: Chhattisgarh, Madhya Pradesh, Uttar Pradesh, East, Bihar, Jharkhand, Odisha, West Bengal; northeast region: Arunachal Pradesh, Assam, Manipur, Meghalaya, Mizoram, Nagaland, Sikkim, Tripura; west region: Dadra and Nagar Haveli, and Daman and Diu, Goa, Gujarat, Maharashtra; and south region: Andaman and Nicobar Islands, Andhra Pradesh, Karnataka, Kerala, Lakshadweep, Puducherry, Tamil Nadu, Telangana. Socio-economic status was measured through a composite wealth index. Other predictor variables used in the analysis included whether the respondent has had children in the past (parity or children ever born), has a desire to have children, her current contraceptive method, whether her current pregnancy was wanted or not, and if any of her pregnancies in the past 5 years ended in abortion.

The source from which ECP was obtained was also analysed among repeated users of ECP, those who used ECP at least once, in terms of number of times ECP used in the past 12 months (categorized as 1 time, 2–4 times, and 5 or more times). Source of ECP was recoded into three categories—only private health sector, only public health sector, and mixed. The third category, mixed, represents those who sourced ECP from a combination of public, private or other sources, where ‘other’ signifies relatives, friends, and so on.

### Statistical analysis

The data were analysed using Stata 15 statistical software. For the two survey rounds, bivariate analyses were conducted to understand the awareness (Table 1) and use of ECP (Table 2) in relation to the different variables described earlier. In Table 1, a two-proportion Z-test is used to test the significance of change in knowledge from 2005–06 to 2019–21. In both Tables 1 and 2, chi-square test is used to test the significance of the association between outcome and independent variables. The significant variables were included in the multivariate analysis, which involved implementing binary logistic regression to examine the determinants of ECP knowledge and use, which is shown in Table 3. Variables representing an episode of abortion in the past 5 years and whether the current pregnancy was wanted were not included in the regression analysis as their sample was much smaller. Only women who reported a termination of pregnancy in their life were asked whether they had had an abortion in the past 5 years and only currently pregnant women could be asked about whether their current pregnancy was wanted or not. Results of multivariate analysis are presented as adjusted odds ratios (OR) with a 95% confidence interval (CI). To observe distribution of source of the ECP among repeat users of ECP, chi-square of association test was used to see whether there is any significant relation between the two.

**Table 1.** Percentage of women having knowledge of emergency contraceptive pills (ECP), across various background characteristics, NFHS 2005–06 and 2019–21, India

	Knowledge		
Characteristics	2005–06 % (Sample)	2019–21 % (Sample)	Change from 2005–06 to 2019–21
**Overall**	**10.75% (124 385)**	**47.61% (724 115)**	**36.86%**
Age group			
15–19	5.96% (23 955)	30.47% (122 480)	24.51%
20–24	10.68% (22 807)	48.43% (118 700)	37.75%
25–29	12.83% (20 653)	55.18% (118 379)	42.35%
30–34	12.82% (17 867)	55.49% (101 049)	42.67%
35–39	12.84% (16 158)	52.41% (98 068)	39.57%
40–44	11.62% (13 138)	48.96% (81 380)	37.34%
45–49	10.48% (9807)	44.64% (84 059)	34.16%
Place of residence			
Urban	16.14% (56 961)	55.36% (179 535)	39.22%
Rural	8.12% (67 424)	43.88% (544 580)	35.76%
Education level			
No education	5.74% (39 769)	38% (167 304)	32.26%
Primary	9.95% (17 756)	44.33% (84 983)	34.38%
Secondary	13.03% (53 882)	47.99% (370 012)	34.96%
Higher	28.52% (12 966)	62.61% (101 816)	34.09%
Wealth index			
Poorest	4.98% (14 077)	36.99% (149 844)	32.01%
Poorer	6.64% (17 652)	42.01% (160 340)	35.37%
Middle	7.66% (23 682)	46.05% (151 505)	38.39%
Richer	11.97% (30 136)	50.73% (139 607)	38.76%
Richest	20.37% (38 838)	61.28% (122 819)	40.91%
Religion			
Hindu	10.95% (89 957)	47.85% (546 007)	36.9%
Muslim	8.4% (16 742)	45.69% (90 729)	37.29%
Christian	13.96% (10 977)	42.72% (52 146)	28.76%
Sikh	13.91% (2772)	53.32% (16 200)	39.41%
Buddhist	8.88% (1765)	59.84% (9076)	50.96%
Other	15.73% (2172)	49.03% (9957)	33.3%
Marital status			
Never married	6.88% (30 661)	34.58% (181 285)	27.7%
Ever married	11.75% (93 724)	51.67% (542 830)	39.92%
Parity			
0	8.12% (39 776)	38.44% (230 096)	30.32%
1	14.87% (15 144)	58.32% (99 448)	43.45%
2	15.17% (24 554)	53.96% (184 683)	38.79%
3+	9.22% (44 911)	46.13% (209 888)	36.91%
Desire for children			
Spacing	10.97% (22 346)	54.46% (120 851)	43.49%
Undecided	12.65% (1437)	58.17% (23 036)	45.52%
Limiting	13.14% (33 124)	54.6% (183 556)	41.46%
Sterilized/infecund/never had sex	9.46% (67 023)	41.78% (379 553)	32.32%
Current pregnancy wanted			
Yes	10.14% (4415)	53.11% (25 596)	42.97%
No	10.49% (1415)	47.32% (2812)	36.83%
Pregnancy ended in abortion in past 5 years			
No		54.37 (181 190)	
Yes		60.23% (7696)	
Region			
North	13.06% (23 139)	57.14% (147 615)	44.08%
Central	10.92% (22 420)	44.27% (170 002)	33.35%
East	8.81% (18 135)	49.4% (118 357)	40.59%
Northeast	9.13% (21 843)	49.27% (103 433)	40.14%
West	14.31% (16 227)	52.1% (71 841)	37.79%
South	9.07% (22 621)	39.72% (112 867)	30.65%
Current contraceptive method			
Not using	8.45% (71 811)	41.73% (373 721)	33.28%
Pill	17.19% (3503)	62.25% (29 944)	45.06%
IUD	23.9% (2049)	61.53% (13 900)	37.63%
Male condom	23.08% (5923)	67.23% (48 379)	44.15%
Female sterilization	11.22% (32 575)	47.55% (189 021)	36.33%
Traditional method	16.51% (7095)	56.48% (57 293)	39.97%
Other	13.19% (1429)	57.75% (11 497)	44.56%

Note: Percentages are weighted and sample numbers are unweighted. Differences during 2005–6 and 2019–21 are significant at *P* < 0.05 across all the variables. *P* values are obtained by applying Z-test for proportion for comparing percentage distribution of women across survey rounds by subgroups of each of the characteristic.

**Table 2.** Percentage of women who had ever used ECP and those who used ECP twice or more in the past 12 months (frequent user), across background characteristics, NFHS 2019–21, India

Characteristics	Ever user	Frequent user
Total	0.49% (550 273)	0.14% (550 273)
Age group	*P* = <0.000	*P* = <0.000
15–19	0.77% (16 162)	0.10% (16 162)
20–24	0.57% (71 912)	0.14% (71 912)
25–29	0.58% (104 403)	0.16% (104 403)
30–34	0.63% (97 673)	0.17% (97 673)
35–39	0.48% (96 504)	0.09% (96 504)
40–44	0.33% (80 347)	0.08% (80 347)
45–49	0.22% (83 272)	0.05% (83 272)
Place of residence	*P* = <0.000	*P*= 0.035
Urban	0.67% (132 423)	0.13% (132 423)
Rural	0.40% (417 850)	0.11% (417 850)
Education level	*P* = <0.000	*P* = <0.000
No education	0.25% (160 429)	0.07% (160 429)
Primary	0.35% (77 529)	0.09% (77 529)
Secondary	0.31% (251 212)	0.13% (251 212)
Higher	1.07% (61 103)	0.22% (61 103)
Wealth index	*P* = <0.000	*P* = 0.010
Poorest	0.37% (116 844)	0.11% (116 844)
Poorer	0.40% (121 915)	0.10% (121 915)
Middle	0.40% (114 670)	0.11% (114 670)
Richer	0.46% (104 975)	0.10% (104 975)
Richest	0.83% (91 869)	0.16% (91 869)
Religion	*P* = <0.000	*P* = <0.000
Hindu	0.47% (420 413)	0.10% (420 413)
Muslim	0.57% (65 355)	0.18% (65 355)
Christian	0.34% (38 019)	0.10% (38 019)
Sikh	0.67% (12 152)	0.10% (12 152)
Buddhist	0.36% (6724)	0.06% (6724)
Other	0.84% (7610)	0.39% (7610)
Marital status	*P* = <0.000	*P* = <0.000
Never married	5.14% (7443)	0.95% (7443)
Ever married	0.44% (542 830)	0.11% (542 830)
Parity	*P* = <0.000	*P* = <0.000
0	0.84% (56 475)	0.18% (56 475)
1	0.63% (99 366)	0.16% (99 366)
2	0.43% (184 619)	0.10% (184 619)
3+	0.37% (209 813)	0.09% (209 813)
Desire for children	*P* = <0.000	*P* = <0.000
Spacing	0.51% (120 293)	0.14% (120 293)
Undecided	0.67% (22 783)	0.12% (22 783)
Limiting	0.75% (183 471)	0.21% (183 471)
Sterilized/infecund/never had sex	0.14% (206 607)	0.01% (206 607)
Current pregnancy wanted	*P* = 0.114	*P* = 0.007
Yes	0.41% (25 586)	0.06% (25 586)
No	0.33% (2810)	0.17% (2810)
Pregnancy ended in abortion in past 5 years	*P* = <0.000	*P* = <0.000
No	0.49% (181 134)	0.14% (181 134)
Yes	2.50% (7693)	0.64% (7693)
Region	*P* = <0.000	*P* = <0.000
North	0.82% (107 617)	0.15% (107 617)
Central	0.56% (124 646)	0.17% (124 646)
East	0.60% (92 830)	0.13% (92 830)
Northeast	0.89% (78 330)	0.20% (78 330)
West	0.24% (56 621)	0.05% (56 621)
South	0.17% (90 229)	0.05% (90 229)
Current contraceptive method	*P* = <0.000	*P* = <0.000
Not using	0.36% (199 879)	0.07% (199 879)
Pill	1.13% (29 944)	0.46% (29 944)
IUD	0.43% (13 900)	0.06% (13 900)
Male condom	1.30% (48 379)	0.25% (48 379)
Female sterilization	0.14% (189 021)	0.01% (189 021)
Traditional method	0.62% (57 293)	0.08% (57 293)
Other	0.69% (11 497)	0.24% (11 497)

Note: Percentages are weighted, and numbers are unweighted; chi-square test of association is used to see association between ever user or frequent user and several characteristics, *P* < 0.01 represents statistical significance at 1% level.

**Table 3.** Adjusted odds ratio of knowledge and ever use of ECP by different background characteristics, estimates from binary logistic regression (multivariate analysis), NFHS 2019–21, India

	Adjusted odds ratio (95% confidence interval)	
Characteristics	Knowledge of ECP	Ever use of ECP
Age group		
15–19	Ref.	Ref.
20–24	1.35 (1.33, 1.38)***	1.24 (0.89, 1.73)
25–29	1.60 (1.56, 1.63)***	1.58 (1.13, 2.20)***
30–34	1.70 (1.66, 1.75)***	1.73 (1.23, 2.43)***
35–39	1.65 (1.61, 1.70)***	1.58 (1.12, 2.24)**
40–44	1.54 (1.50, 1.58)***	1.22 (0.85, 1.75)
45–49	1.39 (1.35, 1.43)***	1.01 (0.70, 1.47)
Type of place of residence		
Urban	Ref.	Ref.
Rural	0.85 (0.84, 0.86)***	0.82 (0.74, 0.91)***
Education level		
No education	Ref.	Ref.
Primary	1.26 (1.23, 1.28)***	1.17 (0.99, 1.39)*
Secondary	1.72 (1.70, 1.75)***	1.44 (1.25, 1.65)***
Higher	2.80 (2.74, 2.86)***	2.01 (1.70, 2.39)***
Wealth index		
Poorest	Ref.	Ref.
Poorer	1.20 (1.18, 1.22)***	1.10 (0.94, 1.29)
Middle	1.38 (1.36, 1.41)***	1.29 (1.10, 1.52)***
Richer	1.50 (1.47, 1.53)***	1.35 (1.14, 1.61)***
Richest	1.85 (1.81, 1.89)***	1.84 (1.53, 2.23)***
Religion		
Hindu	Ref.	Ref.
Muslim	0.92 (0.91, 0.94)***	1.05 (0.93, 1.19)
Christian	0.87 (0.84, 0.90)***	0.75 (0.51, 1.09)
Sikh	0.70 (0.67, 0.72)***	0.76 (0.56, 1.04)*
Buddhist	1.43 (1.34, 1.52)***	1.06 (0.53, 2.14)
Other	0.89 (0.83, 0.95)***	0.99 (0.58, 1.69)
Marital status		
Never married	Ref.	Ref.
Ever married	1.84 (1.77, 1.91)***	0.35 (0.09, 1.35)
Children ever born		
0	Ref.	Ref.
1	1.07 (1.04, 1.09)***	1.05 (0.87, 1.25)
2	1.00 (0.97, 1.02)	0.94 (0.77, 1.14)
3+	0.94 (0.92, 0.97)***	1.10 (0.89, 1.37)
Desire for children		
Spacing	Ref.	Ref.
Undecided	1.11 (1.07, 1.15)***	1.18 (0.95, 1.46)
Limiting	0.98 (0.96, 1.00)*	1.23 (1.07, 1.40)***
Sterilized/infecund/never had sex	0.99 (0.95, 1.02)	0.58 (0.39, 0.86)***
Region		
North	Ref.	Ref.
Central	0.70 (0.69, 0.71)***	0.87 (0.76, 0.99)**
Eest	0.94 (0.92, 0.96)***	1.14 (0.99, 1.30)*
Northeast	0.87 (0.84, 0.89)***	1.37 (1.11, 1.69)***
West	0.74 (0.72, 0.75)***	0.36 (0.30, 0.44)***
South	0.44 (0.43, 0.45)***	0.37 (0.30, 0.45)***
Current contraceptive method		
Not using	Ref.	Ref.
Pill	1.60 (1.55, 1.64)***	2.56 (2.20, 2.98)***
IUD	1.39 (1.34, 1.45)***	1.08 (0.80, 1.46)
Male condom	1.62 (1.59, 1.66)***	2.35 (2.07, 2.67)***
Female sterilization	1.13 (1.10, 1.18)***	1.05 (0.70, 1.57)
Traditional method	1.24 (1.21, 1.26)***	1.45 (1.26, 1.67)***
Other	1.50 (1.43, 1.56)***	2.04 (1.57, 2.64)***
Constant	0.26 (0.25, 0.27)***	0.00 (0.00, 0.02)***

Note: *P < 0.1, **P < 0.05, ***P < 0.01; Ref. reference category.

## Results

### Awareness of emergency contraceptive pills in India

For awareness of ECP, the bivariate analysis is shown in Table 1, whereas the multivariate analysis is shown in Table 3. A total of 11% of surveyed women in 2005–06 (aged 15–49 years) had knowledge about ECP, increasing substantially to 48% in 2019–21 (see Table 1). This pattern varied widely according to socio-demographic characteristics. Awareness of ECP was highest among women aged 30–34, increasing from 13% in 2005–06 to 55% in 2019–21. Women living in urban areas were more aware of ECP than their rural counterparts; with these percentages rising markedly over time in both settings (awareness in urban areas: 16% in 2005–06 to 55% in 2019–21, and awareness in rural areas: 8% in 2005–06 to 44% in 2019–21). Awareness of ECP increased with higher education and wealth status. In 2019–21, around one third of women in the poorest wealth quintile were aware of ECP, with this level being close to two-thirds among the richest quintile.

Even though Buddhism is practiced by a very small proportion of the population in India, knowledge among Buddhist women was the highest compared to women observing other religions in 2019–21. There was a dramatic change over time, as only 9% of Buddhist women were aware of ECP in 2005–06, but 60% in 2019–21. Married women were much more knowledgeable than never-married women (51% and 35%, respectively). Among currently pregnant women, 53% of the women who did not want their current pregnancy were unaware of ECP while 47% of those who did want their current pregnancy were aware. A total of 60% of the women whose most recent pregnancy ended in abortion knew about ECP.

**Figure 1.**
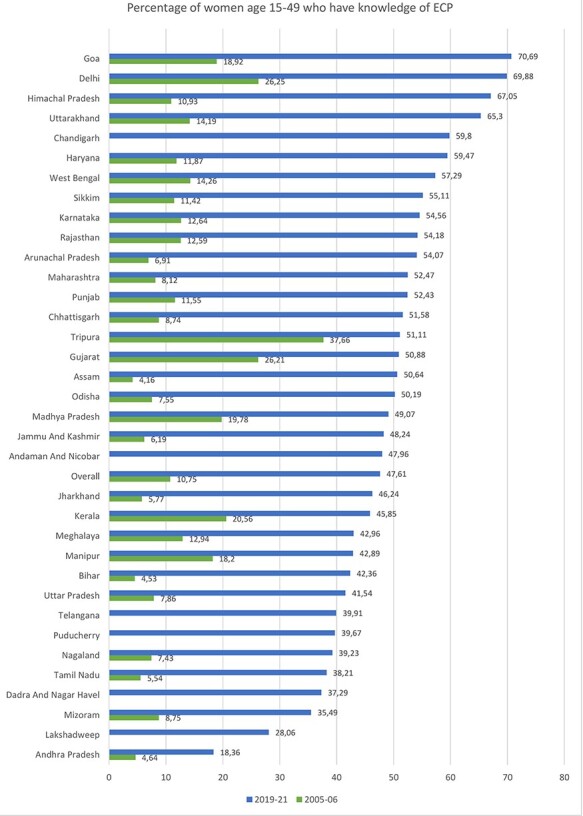
Knowledge of ECP by State/UT in India

Knowledge about ECP varied across the country, with women residing in the northern Indian regions being most aware of ECP (57% in 2019–21) while those in the south were the least aware (40% in 2019–21). As shown in Fig. 1, Goa (71%) and Delhi (70%) showed the highest proportion of women knowing about ECP in 2019–21, whereas the lowest level of knowledge was observed in Andhra Pradesh (<20%).

The same findings are observed through the multivariate analysis (Table 3). Age, place of residence, education level, wealth status, religion, marital status, region and current contraceptive method were significantly related to knowledge of ECP in 2019–21.

### Use of emergency contraceptive pills in India

**Figure 2.**
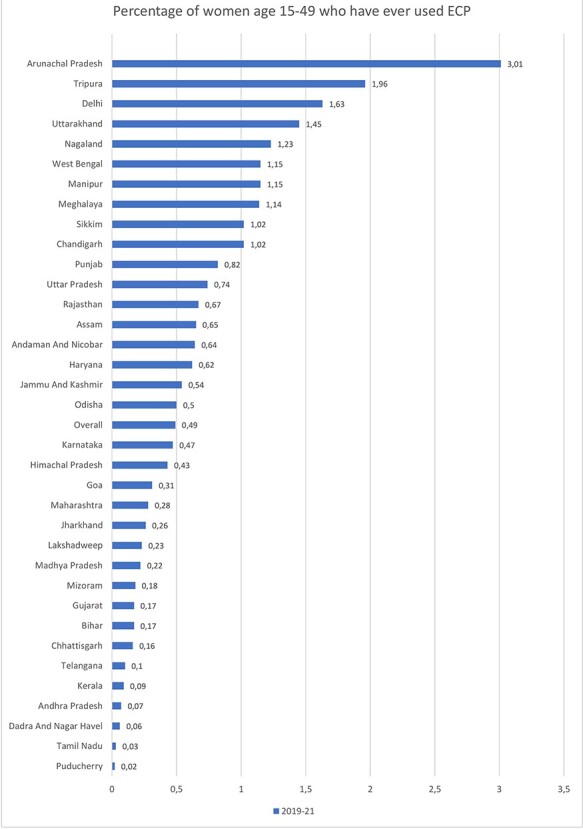
User of ECP by State/UT in India

For use of ECP, the bivariate analysis is shown in Table 2, whereas the multivariate analysis is shown in Table 3. The overall prevalence of ever use of ECP was very low (0.49%). However, it varied according to the different socio-demographic characteristics of the survey respondents (see Table 2). Young women aged 15–19 years reported using ECP to a larger extent (0.77%) than women in the other age groups. The use of ECP was more common in urban women (0.67%) than in rural women (0.40%). The highest share of users was found among women in the most affluent wealth quintile (0.83%) and those with higher education (1.07%). The prevalence of ECP users among unmarried women (5.14%) was around 10 times higher than among married women (0.44%). Those women who wanted to limit their fertility (0.75%) were using ECP more than those who wanted to space their pregnancies (0.51%). Highest usage was found in women who had no children (0.84%) and it decreases as the number of children ever born increased. About 2.5% of women who had had an abortion in the past 5 years had ever used ECP. Women whose current contraceptive method was condoms had a higher prevalence of ECP use (1.30%) than women who used other methods. Table 2 also shows the prevalence for those who used ECP twice or more in the past 12 months (frequent user), which was lower (0.14%) than that of ever user. However, the pattern across all the background characteristics was similar to that of ever user. Amongst regions, the proportion of ECP ever users was highest among women in the northeast region (0.89%) and lowest among women in the southern regions (0.17%) of India. Figure 2 shows women in Arunachal Pradesh (3.01%) had the highest prevalence, and Puducherry (0.02%) had the lowest proportion of ECP use.

While the bivariate analysis in Table 2 shows that there is an association between ECP use and age, marital status, having had children previously and religion, the relationship was not significant in the multivariate analysis (see Table 3). Place of residence, education, wealth index, preference to have children, region and current contraceptive method were significantly associated with using ECP. Those who had higher education were twice as likely to use ECP than women who had no education [AOR: 2.01; CI: 1.70–2.39]. The odds of ECP use by women in rural areas were lower than those in urban areas [AOR: 0.82; CI: 0.74–0.91]. The likelihood of ECP use increased as the wealth index rose. Women who wanted to limit their fertility were 23% more likely to use ECP than those who wished for spacing [AOR: 1.23; CI: 1.07–1.40]. Women who were using oral pills as their current contraceptive method were around three times more likely to use ECP than women who were not using any contraceptive method [AOR: 2.56; CI: 2.20–2.98].

### Source of ECP among repeated users of ECP

Among those who used ECP once or more, the distribution of sources from where it was obtained (type of outlet) was also studied (see Table 4). ECP was typically obtained from private health facilities (60%) and less frequently from public facilities (23%) and other sources such as shops, outlets or supplied by relatives. There was a statistically significant association between the source from which ECP is obtained and the frequency of use. Still, women who took a higher number of pills tended to switch from private to public health facilities in order to maintain their ECP supply.

**Table 4.** Distribution of number of times ECPs used in the past 12 months with respect to the source it was accessed from among repeat users of ECP who used ECP at least once, NFHS 2019–21, India

	Times used emergency contraception in past 12 months			
ECP source	1	2 to 4	5 or more	Total
Only public	19.04	23.52	27.34	22.78
Only private	63.62	60.00	55.19	60.24
Mix	17.34	16.48	17.47	16.99
Total	100	100	100	100

Note: The percentages are weighted. Relationship between source and repeated use showed significant association with *P* value = 0.000 using chi-square test of association.

## Discussion

Given the continued high prevalence of unwanted pregnancies and the low and inconsistent use of modern contraception in India, it is important that the population’s access to a variety of contraceptive methods be promoted. The current study sought to examine ECP awareness, use and variation across the population and over time, as there is limited evidence in this area. Understanding who ECP users are and who does not know about or use ECPs can enable better targeting of policies seeking to improve reproductive health in India.

Our study confirms a significant gap in knowledge and infrequent use of ECP in India. Even after 15 years of legalization and wide availability of ECP, more than half of the women are unaware of this contraceptive method, with less than 1% having ever used ECP. Our findings are consistent with other studies from India that found that the odds of having heard of or used the emergency contraception method generally increase with wealth and literacy level (Takkar *et al.*, 2005).

While this analysis found that women aged 30–34 years are more aware of ECP, another study conducted in three cities of central India found that younger women are three times more aware of ECP than those aged 30 and above (Kumar *et al.*, 2007). Younger unmarried couples may be more likely to use ECP because of high fertility and a strong preference to delay pregnancy (Jejeebhoy, 1998). In addition, our research shows the use of ECP to be higher among adolescents, which is in agreement with another study on ECP in India (Rocca *et al.*, 2013). Younger and poorer women experience higher rates of contraceptive failure and are least able to cope with an unexpected pregnancy, obtain maternal health care and access safe abortion services (Black *et al.*, 2010; Bradley *et al.*, 2019).

This study makes a significant contribution in the literature by showing knowledge of ECP across two time periods in India and how various background characteristics affect the awareness and use of ECP using a nationally representative dataset for women aged 15–49 years that had not been studied before. It analysed ECP across individual characteristics such as desire for children, parity and current method of contraceptive, which can help understand the users and non-users of ECP using the most recent data available. Our study revealed that condom and pill users are more likely to use ECP than other method users. While condom and pill users are likely to be motivated to avoid pregnancy, both condoms and pills have higher potential for incorrect or inconsistent use compared to surgical sterilization (the most popular form of modern contraception in India) (Bradley *et al.*, 2019). Those using traditional contraceptive methods (predominantly withdrawal) may be less motivated to avoid pregnancy or less willing to use modern methods in general, including ECP.

Awareness and use of ECP vary across India’s states and union territories. Knowledge and use of ECP are lowest in the southern states of India. The southern states have higher levels of female literacy and empowerment, so it is surprising to find low knowledge and use of ECPs. This finding may be partially driven by the fact that Tamil Nadu, a large southern state, has banned the advertisement and sale of ECP over the counter since 2006 based on moral grounds (Shukla and Arends-Kuenning, 2017). Higher rates of awareness and use in other parts of India may be partially driven by the presence of large urban centres such as Delhi; high ECP sales have been found in metro cities (Takkar *et al.*, 2005; Dixit *et al.*, 2015; Mehta *et al.*, 2020). The highest prevalence of ECP is found in Arunachal Pradesh, and more research is needed to understand why this is the case, as the state has the largest tribal population in India, comprising various matrilineal tribes.

The most common source of ECP in India is observed to be the private health sector, which is also found in our research (Takkar *et al.*, 2005; Kumar *et al.*, 2007). Knowledge and use of ECP in 2019–21 remained lower in rural areas than in urban areas, with a prevalence of only 43% of ECP awareness and 0.40% of ever use. Community-level distribution of ECP by ASHAs was approved in 2011 (Kumar *et al.*, 2007) and the National Rural Health Mission has tried to reach women in their homes in rural areas (Shukla and Arends-Kuenning, 2017). Still, the current study shows that only 22% of women who have accessed ECP did so through the public sector. Frontline workers may have struggled to take on education and provision of the ECP due to their already heavy workload, insufficient training on the ECP, or supply chain issues leading to ECP stock outs. Some studies have shown that even ASHAs are unaware of the correct use of the method and lack counselling skills, which could be the reason for the low prevalence of ECP use in rural areas (Kumar *et al.*, 2007; Dixit *et al.*, 2015). There have also been reports of misbelief and confusion between oral contraceptive pills, ECP and abortion pills among the ASHAs and ANMs (Dixit *et al.*, 2015). Another possible explanation is inadequate incentives for frontline workers working to increase awareness of diverse contraceptive methods (Kumar *et al.*, 2007).

In India, in addition to low awareness, the stigma around sex and negative beliefs about emergency contraception may also reduce use. There are misconceptions that ECP is an abortifacient, leading to its under-utilization (Verma *et al.*, 2021). ECP use is sometimes associated with extra-marital or pre-marital sex, both of which are highly taboo and may limit the accessibility of ECP (Purohit *et al.*, 2013). A qualitative study in Uttar Pradesh found that health-care providers think that sexual activity among young girls has increased due to the easy availability of ECP (Mehta *et al.*, 2020). Still, studies eliciting detailed information in this area are scarce.

This study had some limitations. ECP use among sexually active women remains relatively rare. Therefore, this study cannot examine specifics regarding users of ECP in further depth or at the sub-national level due to the small number of women that have ever used ECP. It could be possible that women are afraid to report usage of ECP as it is associated with negligence in using contraception, leading to a reporting bias during the collection of data. Given that use of oral contraceptive pills and medical abortion pills is higher than reported use of ECPs, some respondents may have been confused between the three types of pills, which could lead to under-reporting of ECP use. Recall bias could arise too as a woman might not remember how many ECPs she has taken in the past 12 months. Although one can keep these issues in mind but the estimates might not get affected much till the biases are fairly random (Boerma and Sommerfelt, 1993). Qualitative studies or research studying transition patterns from use of one method to another family planning method could also help in understanding when the user wishes to use ECP. There is a lack of data on the awareness of health-care providers, which could play an important role in enhancing the present research.

## Conclusion

The present study shows that awareness of ECP was lower among women with unwanted pregnancies. Hence, to prevent women from the strain of abortion and unwanted pregnancies, increased population-level awareness of ECP is needed. To achieve this, ASHAs need support and training to guide women through their contraceptive options, including choices in case of contraceptive failure (Kumar *et al.*, 2020). Media campaigns (including through print, television and social media) and patient–provider interaction can increase awareness about ECP and address stigma and misinformation about its use. These efforts should specifically target adolescents, women in rural areas and poorer women, given their lower awareness compared to older, urban and wealthier women. While some states in India have a higher awareness than others, all states remain far from 100% awareness and could thus all benefit from these campaigns.

## Supplementary Material

czad049_Supp

## Data Availability

The data underlying this article are available in the DHS Program, available on request from https://dhsprogram.com/data/.

## Abbreviations

ECP  Emergency Contraception Pill

NFHS  National Family Health Survey

ASHA  Accredited Social Health Activist

ANM  Auxiliary Nurse and Midwife

Cu-IUD  Copper Intrauterine Device

WHO  World Health Organization

MoHFW  Ministry of Health and Family Welfare

AOR  Adjusted Odds Ratios

CI  Confidence Interval

OCP  Oral Contraceptive Pill

## Reflexivity statement

All four co-authors for this paper are women. Renu and Pooja are based in India. Renu is an early career researcher in the field of reproductive health. Pooja is a doctoral research scholar with maternal and child health as the focus of her research. Dina and Kerry are based in the UK and Canada, respectively. Dina has been involved in research in health systems and policy for close to two decades now. Kerry lived in India for a decade and has over 12 years of experience in health policy and systems research.
