# Supplementary material for: Do Indian women know about and use the emergency contraceptive pill? An analysis of nationally representative data from 2005–06 and 2019–21
Source: Health Policy Plan. 2023 Nov 16;38(Suppl 2):ii51–61. doi: 10.1093/heapol/czad049 (PMC10679928; doi:10.1093/heapol/czad049)
Supplement: czad049_Supp [file czad049_supp.zip › suppl_data/Table S1_clean version.docx]

**Supplementary Table**

Table S1: Percentage distribution of women (15–49 years) by selected demographic and socioeconomic characteristics, India, 2005–16 (Sample Description).

| **Characteristics** | **2005-06** | **2019-21** |
| --- | --- | --- |
| **Age group** | |  |
| 15-19 | 19.95 | 16.92 |
| 20-24 | 18.31 | 16.49 |
| 25-29 | 16.41 | 16.18 |
| 30-34 | 14.19 | 13.86 |
| 35-39 | 12.76 | 13.47 |
| 40-44 | 10.49 | 11.29 |
| 45-49 | 7.88 | 11.77 |
| **Place of Residence** | |  |
| Urban | 32.82 | 32.49 |
| Rural | 67.18 | 67.51 |
| **Education Level** | |  |
| No education | 40.59 | 22.43 |
| Primary | 14.70 | 11.73 |
| Secondary | 37.41 | 50.18 |
| Higher | 7.30 | 15.65 |
| **Wealth Index** | |  |
| Poorest | 17.46 | 18.50 |
| Poorer | 18.99 | 20.00 |
| Middle | 20.17 | 20.52 |
| Richer | 20.99 | 20.81 |
| Richest | 22.40 | 20.17 |
| **Religion** |  |  |
| Hindu | 80.52 | 81.36 |
| Muslim | 13.62 | 13.48 |
| Christian | 2.45 | 2.35 |
| Sikh | 1.79 | 1.57 |
| Buddhist | 0.81 | 0.63 |
| Other | 0.81 | 0.61 |
| **Marital Status** | |  |
| Never Married | 20.47 | 23.76 |
| Ever Married | 79.53 | 76.24 |
| **Parity** |  |  |
| 0 | 28.63 | 30.81 |
| 1 | 11.92 | 14.25 |
| 2 | 19.71 | 26.99 |
| 3+ | 39.74 | 27.95 |
| **Region** |  |  |
| North | 13.28 | 14.11 |
| Central | 23.26 | 24.89 |
| East | 22.44 | 22.76 |
| Northeast | 3.95 | 3.69 |
| West | 14.83 | 14.09 |
| South | 22.24 | 20.45 |
| **Current contraceptive method** | | |
| Not using | 56.16 | 49.88 |
| Pill | 2.31 | 3.69 |
| IUD | 1.30 | 1.53 |
| Male condom | 3.93 | 7.03 |
| Female sterilization | 29.55 | 29.05 |
| Traditional method | 5.59 | 7.45 |
| Emergency contraception | 0.00 | 0.04 |
| Other | 1.16 | 1.34 |
| **Total number (unweighted)** | **124385** | **724115** |

Note: Percentages are weighted
